# Metoclopramide-Induced Hyperprolactinemia Mimicking Prolactinoma in a Young Woman Receiving Fragmented Care: A Case Report

**DOI:** 10.7759/cureus.112043

**Published:** 2026-07-04

**Authors:** Mohammad Ballout, Mark Schury

**Affiliations:** 1 Family Medicine, McLaren Oakland Hospital, Pontiac, USA

**Keywords:** amenorrhea, case report, dopamine antagonist, health literacy, hyperprolactinemia, medication reconciliation, metoclopramide, polycystic ovary syndrome, prolactinoma

## Abstract

Hyperprolactinemia accompanied by headache and visual symptoms commonly prompts pituitary imaging to rule out prolactinoma. Several medications can elevate serum prolactin into ranges that overlap with prolactin-secreting adenomas, and metoclopramide, a dopamine D2 receptor antagonist used for gastroparesis and nausea, is among the most frequent offenders. Headache and blurred vision are also listed as adverse effects, reproducing the symptomatic triad clinicians associate with a pituitary tumor. We describe a 22-year-old woman with polycystic ovary syndrome who presented with a prolactin level of 160.3 ng/mL, intermittent blurry vision, and chronic headaches managed with onabotulinumtoxinA through neurology. Pituitary MRI was unremarkable. Despite repeated direct questioning, her initial medication history included only losartan. Metoclopramide was identified through deliberate review of outside gastroenterology records, where it had been prescribed for chronic constipation. One month after discontinuation, prolactin levels had returned to normal at 11.2 ng/mL. Menstrual function showed initial improvement, as she experienced her first menstrual cycle after approximately one year of amenorrhea. However, regular menses did not fully resume; cycles remained irregular, likely in the setting of her underlying polycystic ovary syndrome (PCOS). This case illustrates how drug-induced hyperprolactinemia can be missed when the medication list is incomplete, particularly in fragmented care settings, and how deliberate review of outside documentation can spare patients unnecessary imaging.

## Introduction

The diagnostic workup for hyperprolactinemia depends heavily on one early step: identifying whether a medication is responsible. Endocrine Society guidelines recommend excluding pharmacologic causes before ordering pituitary imaging [1]. When the medication list is accurate, this is straightforward. When it is not, imaging is pursued for a problem that could have been resolved by stopping a prescription. Reports of metoclopramide-induced hyperprolactinemia frequently feature this gap, but the gap itself is rarely examined closely. The following case demonstrates how an incomplete medication list, in a patient receiving care across multiple disconnected practices, produced a clinical picture indistinguishable from prolactinoma and was resolved only through review of outside referral documentation.

## Case presentation

Patient information

A 22-year-old woman with a history of polycystic ovary syndrome (PCOS) presented to our family medicine clinic for follow-up of headaches, intermittent blurry vision, irregular menses, and an elevated prolactin level obtained during a prior workup. She reported approximately one year of amenorrhea, broken by a single heavy four-day menses. She denied galactorrhea. Her headaches had been longstanding and were managed with onabotulinumtoxinA injections through neurology.

At the initial visit, she reported taking losartan, a multivitamin, and vitamin B12. Combined oral contraceptives had previously been discontinued because of weight gain and worsening headaches. She was sexually active and preferred to avoid hormonal contraception. Allergies were not reported.

Gastrointestinal history

Separately, and under the care of a different provider, the patient had been referred to gastroenterology for IBS-type symptoms with chronic constipation. A trial of linaclotide (Linzess) provided no relief, and metoclopramide was subsequently started with reported benefit. This medication was not disclosed during our initial intake, and the prescribing relationship existed entirely outside our clinic.

Social determinants and care context

This patient received care in an underserved setting with multiple providers across separate health systems and no shared electronic medical record. The pattern of non-disclosure was consistent with limited health literacy and system-navigation barriers rather than intentional withholding; the patient did not consider a medication prescribed by another specialty for a gastrointestinal complaint to be relevant to a visit focused on headaches and menstrual irregularity.

Clinical findings

Focused neurologic and ophthalmic examinations were non-focal. No visual field deficit was documented.

Timeline of events

The patient's symptoms began approximately one year before presentation. Six to eight months prior, she had been evaluated for chronic constipation and referred to gastroenterology, where metoclopramide 10 mg was started four to six months before presentation after linaclotide failed. One month before the presentation, endocrine evaluation revealed an elevated prolactin level, and a pituitary MRI was obtained. On day seven of our evaluation, metoclopramide was identified on outside referral documentation and discontinued. Repeat prolactin obtained on day 30 demonstrated normalization.

Diagnostic assessment

The endocrine workup at presentation and at follow-up is summarized in Table 1.

**Table 1:** Laboratory values. ALT, alanine aminotransferase; AST, aspartate aminotransferase; DHEA, dehydroepiandrosterone; TSH, thyroid-stimulating hormone. Initial endocrine evaluation revealed an elevated prolactin with associated hormonal findings summarized in this table. Follow-up prolactin one month after metoclopramide discontinuation had normalized.

Parameter (units)	Patient value	Reference range
Prolactin, initial (ng/mL)	160.3 (H)	3.0-30.0 (non-pregnant female)
Prolactin, follow-up (one month later) (ng/mL)	11.2	3.0-30.0 (non-pregnant female)
Luteinizing hormone (mIU/mL)	12.5	Follicular 1.9-12.5; Mid-cycle 8.7-76.3; Luteal 0.5-16.9
Follicle-stimulating hormone (mIU/mL)	3.0	Follicular 2.5-10.2; Mid-cycle 3.1-17.7; Luteal 1.5-9.1
Estradiol (pg/mL)	276	Follicular 19-144; Mid-cycle 64-357; Luteal 56-214
Progesterone (ng/mL)	1.4	Follicular <1.0; Luteal 2.6-21.5
Total testosterone (ng/dL)	74 (H)	2-45
DHEA-sulfate (mcg/dL)	349	14-349
TSH (mIU/L)	1.33	0.4-4.5
AST (U/L)	21	10-30
ALT (U/L)	13	6-29
Creatinine	0.91 mg/dl	0.5-0.96

**Figure 1:**
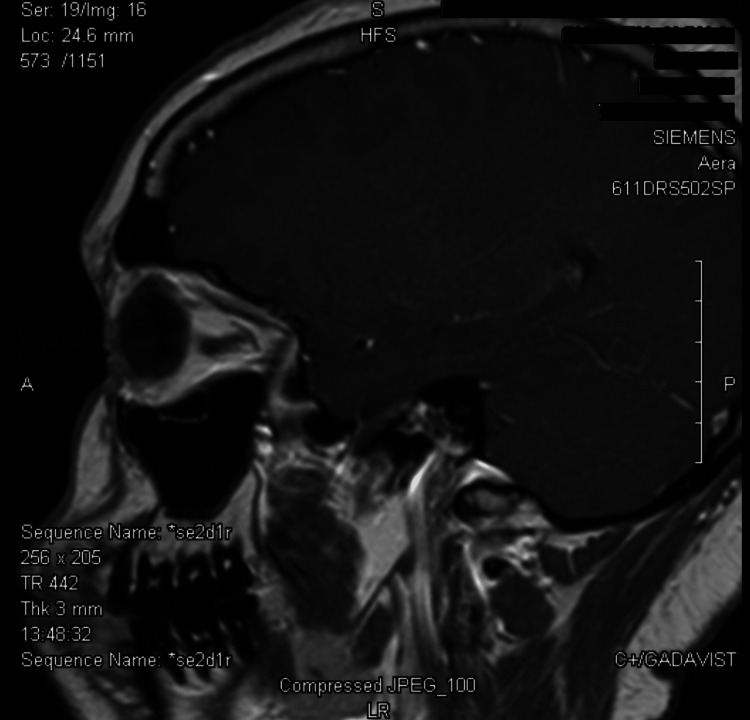
Pituitary MRI with contrast demonstrating a normal-sized gland with homogeneous enhancement and no evidence of adenoma.

Pituitary MRI with contrast using a dedicated pituitary protocol demonstrated a normal-sized gland with homogeneous enhancement, minimal leftward infundibular deviation, an intact optic chiasm, and no enhancing lesion or restricted diffusion (Figure 1). The radiology impression was no evidence of micro- or macroadenoma and no acute intracranial pathology.

With a normal MRI and an unexplained prolactin elevation, identification of metoclopramide on outside records redirected the diagnostic reasoning toward a pharmacologic etiology. Prolactinoma was considered less likely given the normal MRI and the subsequent clinical course. PCOS-related anovulation contributed to menstrual irregularity but would not account for the degree of prolactin elevation observed. Pregnancy, hypothyroidism, macroprolactinemia, and renal or hepatic dysfunction were not supported by the clinical picture.

Therapeutic intervention

Metoclopramide was discontinued in coordination with the patient's gastroenterologist. She was counseled on the identified etiology and on the importance of reporting all medications, including those prescribed by other providers, at every visit. PCOS management continued through gynecology, headache care through neurology, and routine ophthalmic follow-up was advised.

Follow-up and outcomes

One month after metoclopramide cessation, serum prolactin returned to within the normal range and menstrual regularity improved. No further imaging was pursued.

## Discussion

Prolactin secretion from anterior pituitary lactotrophs is tonically inhibited by dopamine acting on D2 receptors [2]. Any agent that blocks this inhibition can raise serum prolactin, and dopamine receptor antagonists account for most cases of drug-induced hyperprolactinemia [3]. Metoclopramide is a particularly potent offender because it is a D2 antagonist that readily crosses the blood-brain barrier [4]. Reported prolactin elevations on metoclopramide can exceed 200 ng/mL, well within the range seen with microprolactinomas [3,5]. Headache and visual disturbance are listed as adverse effects on the drug's label [6], reproducing the symptomatic triad clinicians most often associate with a prolactin-secreting tumor.

In this patient, that mimicry was reinforced by an additional layer of misdirection. Her chronic headaches predated metoclopramide exposure and were already being managed with onabotulinumtoxinA through neurology, which drew attention toward an established headache disorder rather than a new medication-related complaint. The blurry vision likewise had a seemingly independent trajectory, completing the triad without raising suspicion of a side effect.

The diagnosis ultimately failed to surface through standard medication review. Repeated direct questioning produced only losartan, not because the patient withheld information but because she did not recognize a medication prescribed by gastroenterology for a gastrointestinal complaint as relevant to a visit focused on headaches and menstrual irregularity. This pattern is well documented in safety-net populations, where care fragmentation, multiple prescribers across disconnected records, and variable health literacy compound one another [7,8]. The answer was ultimately found by reviewing the gastroenterology referral documentation directly, a step that is straightforward in principle but easily deferred under time pressure. When outside records are unavailable, clinicians can use practical fallback methods such as pharmacy reconciliation, prescription-monitoring database review when appropriate, asking patients to bring medication bottles or photos of labels, or scheduling a focused follow-up medication review before moving to imaging.

When prolactin is elevated and the reported medication list contains no known offender, it is reasonable to go beyond the patient's self-report. Pharmacy records, outside provider notes, and specific questioning about medications for gastrointestinal, psychiatric, or pain indications often identify dopamine antagonists that would otherwise be missed [1,3]. Earlier recognition allows a drug-washout trial to precede imaging, sparing patients both cost and anxiety.

## Conclusions

Metoclopramide-induced hyperprolactinemia can closely reproduce the clinical picture of prolactinoma, and the diagnosis depends on recognizing the exposure. When the medication list is incomplete, that recognition fails. In fragmented care environments, a standard medication review may not be sufficient; asking patients to name every physician they see and what each has prescribed can surface medications they would not otherwise mention. When hyperprolactinemia remains unexplained, a thorough medication review should accompany the diagnostic evaluation and may help avoid unnecessary imaging.
